# Diagnosis and Surgical Management of Nonsyndromic Nine Supernumerary Teeth and Leong's Tubercle

**DOI:** 10.1155/2016/8641867

**Published:** 2016-03-15

**Authors:** Christiane V. Cruz, Andrea L. Soares, David N. Braga, Marcelo C. Costa

**Affiliations:** ^1^Department of Pediatric Dentistry and Orthodontics, Federal University of Rio de Janeiro, Brazil; ^2^Department of Oral Biology, Fluminense Federal University, Brazil; ^3^Department of Maxillofacial Surgery, Federal University of Rio de Janeiro, Brazil

## Abstract

Nonsyndromic multiple supernumerary teeth (ST) and Leong's tubercle are a condition with a very low prevalence and a multidisciplinary approach is required to restore function and aesthetics. So, this case report aimed at presenting a rare case of nonsyndromic nine supernumerary teeth and Leong's tubercle in a pediatric patient, without any evident familial history, showing its diagnosis and surgical management.

## 1. Introduction

Supernumerary teeth (ST) are a numeric dental anomaly characterized by the formation of teeth in excess of the normal dental formula, occurring in both the primary and permanent dentition [1, 2]. The etiology of ST is still unknown. A number of theories have been postulated to try to explain their presence, including atavism (evolutionary throwback), tooth germ dichotomy, genetic and environmental factors, and hyperactivity of the dental lamina. However, all theories are hypothetical due to the inability to obtain sufficient embryologic material on their origin [3]. Their frequency ranges from 0.3% to 3.6% [4, 5] and they are two times more common in males than females [5, 6]. Multiple ST are usually associated with syndromes such as cleidocranial dysplasia and Gardner's syndrome [7]. However, multiple ST in nonsyndromic patients is a rare condition [8] and less than 1% of cases are reported [9, 10]. Nonetheless, teeth that remain unerupted may cause aesthetic and functional problems due to overretained primary teeth, delayed or ectopic eruption of permanent, displacement and rotation of adjacent teeth, crowding, development of diastema, crossbite [10, 11], eruption into the floor of the nasal cavity [12], and root resorption of adjacent teeth [10].

Accessory cusps are variations of tooth shape and their frequency varies depending on the type and the tooth affected. The most commonly reported accessory cusps are cusp of Carabelli of the molars that represents 68% [13], 8% for Leong's tubercle of premolars, and between 1% and 7.7% for the talons cusps of the incisors [14]. The clinical aspects include a cusp like accessory structure varying in size from a prominent cingulum to a marked projection affecting the enamel surface of the teeth [15]. Regarding clinical disturbances, it may cause occlusal interferences, esthetic disturbances, accidental cusp fracture leading to loss of pulp vitality, irritation of tongue during speech and mastication, nursing difficulties, caries, and displacement of the affected tooth [16].

The most reliable methods for the diagnosis of supernumerary teeth and accessories cusp are clinical and conventional radiographs (orthopantomogram, periapical and occlusal) and cone-beam computed tomography [7]. Early surgical intervention is the preferred method of treatment to prevent clinical problems and to minimize further complications. To the best of our knowledge, this is the first report that presents a case of multiple supernumerary teeth associated with accessory cusp. So, here we document a case of nine supernumerary teeth in a nonsyndromic pediatric patient and the presence of Leong's tubercle in the premolars usual dental formula, highlighting the diagnosis and surgical management using a multidisciplinary approach. This case report was performed according to The CARE guideline [17].

## 2. Case Report

**Figure 1:**
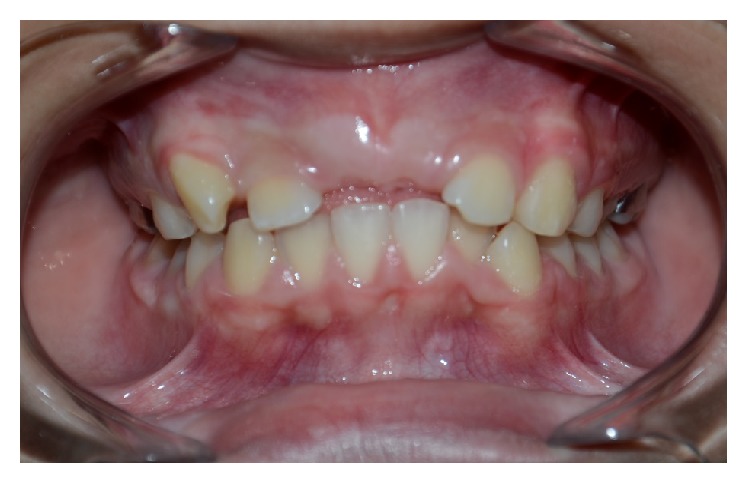
Frontal view showing the absence of the maxillary central incisors.

**Figure 2:**
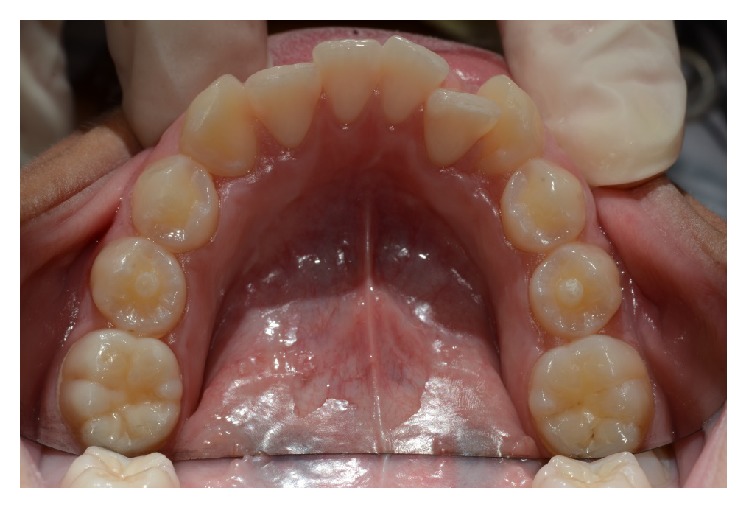
Occlusal view disclosing Leong's tubercle on the second mandibular premolars.

A 10-year-old male, Caucasian patient was referred to the Continuing Education Clinical Program in Pediatric Dentistry at the Federal University of Rio de Janeiro due to the absence of teeth in the maxillary anterior region; this has influenced his social adjustment, impacting on his quality of life (Figure 1). The family's medical and dental history was noncontributory. General physical and extra oral examination did not show any abnormality. An intraoral examination revealed the presence of mixed dentition and the absence of the permanent maxillary central incisors. There was a wide anterior arch space, misalignment of permanent canines and mandibular incisors, and the lack of space for the alignment of the upper permanent incisors. Normal overjet and overbite were observed. The second usual premolars presented Leong's tubercle, which resulted in occlusal interference, performing a premature contact with their antagonists (Figure 2).

**Figure 3:**
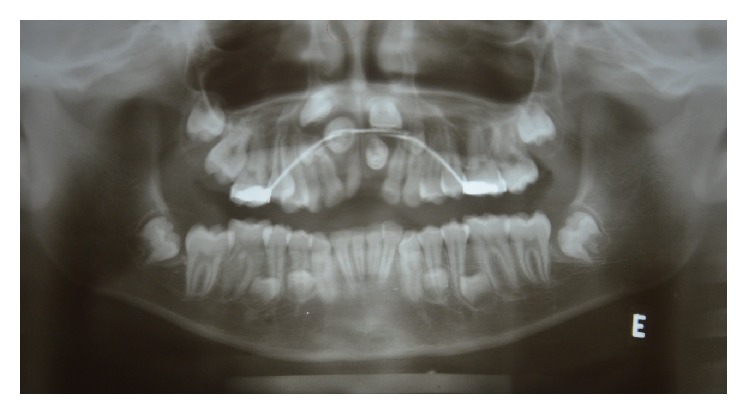
Orthopantomogram. Nine supernumerary teeth distributed in the maxillary and mandibular arches.

The analysis of the orthopantomogram and occlusal radiographs revealed the presence of nine impacted supernumerary teeth in the four quadrants, distributed as follows: in the maxilla there were one conical and two tuberculate mesiodens and two supplemental teeth in the posterior segment. In the mandible, there were four tuberculate teeth arranged in the premolar region, two in the right side and the other ones on the left side (Figure 3). The cone-beam computed tomography showed that the central incisors were arranged horizontally in the floor of the nasal cavity and presented a complete root development.

**Figure 4:**
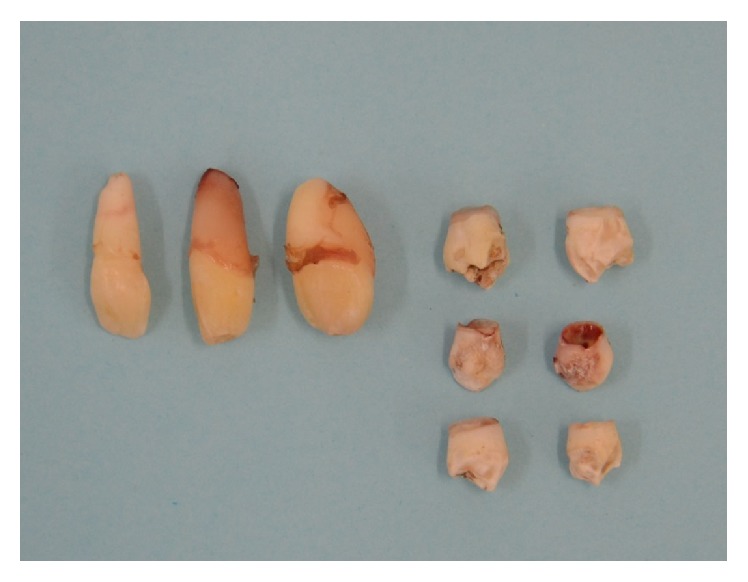
Nine supernumerary teeth.

After a thorough diagnosis by the multidisciplinary team, pediatric dentistry, oral surgeon, and orthodontist, by analyzing the orthopantomogram and cone bean computed tomography as well as his systemic condition, the treatment plan was defined. The treatment of choice for Leong's tubercle was selective grinding. It was performed using a diamond stern conical drill under water to eliminate premature contact. The nine ST were extracted in a single surgical time under general anesthesia (Figure 4). The orthodontic traction of the superior central incisors was proposed aiming to their alignment in the upper arch. However, there was not enough space to carry out the tooth alignments in both the upper and lower arches. Thereby, we proposed the extraction of four premolars in order to obtain the correct alignment before the referred orthodontic traction, which could improve function and aesthetics. On the other hand, the prognosis of the orthodontic traction was questionable because of the risk of dental ankylosis due to the close contact with the buccal cortical bone. As a result, an additional treatment was proposed, implantology, and/or prosthetic restoration at the end of the growth spurt.

## 3. Discussion

It is essential not only to enumerate but also to identify the ST present clinically and radiographically before a definitive diagnosis and treatment plan can be formulated [9]. In this patient, the ST resulted in a wide anterior arch space because the unerupted mesiodens has caused a retardation or obstruction of eruption of the permanent central incisors which resulted in an aspect of dental absence in the anterior region, impacting the quality of life of the ST patient. The mesiodens in this patient has been probably originated from the permanent dentition tooth bud since in the primary dentition, supernumerary teeth occurred most often in the lateral incisor regions, as opposed to permanent supernumerary teeth, which prevailed in the central incisor regions [18]. Early diagnosis and extraction of mesiodens may prevent malocclusion and dental abnormalities such as delayed eruption, rotation of the permanent incisors and diastema [19, 20]. Teeth located in the nasal cavity are a rare phenomenon but a case has been previously reported in which mesiodens were left untreated and erupted in the nasal cavity [21]. In this patient, it is unlikely that the ST would erupt in the nasal cavity if they were left untreated because of their horizontal position. Nevertheless, it is worth noting that these teeth should be followed up in an attempt to decrease the risk of oral complications.

Classification of ST can be made on the basis of morphology and region [7]. Morphology variations include conical types (small peg-shaped or conical), tuberculate types (with more than one cusp or tubercle frequently described as barrel-shaped and may be invaginated), and supplemental teeth (refers to a duplication of teeth in the normal series). They can be found in almost any region of the dental arch but occur more frequently in the maxilla, especially in the anterior segment. ST may occur singularly or in multiple teeth and unilaterally or bilaterally [9]. Corroborating these findings, we found five ST in the maxilla, of which three were mesiodens (one conical and two tuberculate teeth) and two supplemental teeth which were located on either side of the posterior arch. In addition, we found four ST arranged in the posterior region of the jaw.

Thus in this patient, it was necessary to remove the ST under general anesthesia since the patient was not able to tolerate a long surgical procedure under local analgesia. Furthermore, when surgical removal is indicated, the advantage of avoiding the young children for local analgesia should be kept in mind where about 52% of the patients aged 5 to 10 years often require general anesthesia for removal of ST [22]. It has been advocated that a single surgical time could bring more benefits to the patient and his family has accorded to this decision. ST are associated with disturbances of tooth eruption, midline diastema, or development of a local malocclusion [15]. We decided not to use traction for the two permanent incisors at the same time because their position was unfavorable and the risk of ankylosis as well as their alignment will require the extraction of the first premolars; therefore, we chose conservative management. The right central incisor will undergo traction on a second time under local analgesia, and depending on the performance, the orthodontic plan can be followed up.

Accessory cusps are relatively rare anomalies. The size, shape, and location of these anomalies have wide variations. The central cusp on the occlusal surface of posterior teeth has also been given several descriptions such as supernumerary occlusal cusp, dens evaginatus, premolar odontome, occlusal tubercle, tuberculated premolar, Leong's tubercle, and Leong's premolar [22]. Patients with additional tooth projections should be placed under routine and periodic dental surveillance, which include monitoring of the degree of attrition and tooth vitality. Early diagnosis and management are important if complications are to be prevented. In this report, we performed the enamel grinding on the Leong's tubercle as an attempt to avoid occlusal disturbances such as functional crossbite, premature contact, and accidental cusp fracture to prevent disturbances in the pulp vitality.

Whenever the supernumerary teeth and accessory cusp are diagnosed, a decision with regard to the appropriate management should be made carefully. It is difficult to establish an ideal treatment for these cases. The clinical and radiographic exam is of vital importance to carry out a good treatment.

## 4. Conclusion

The approach to the patients with supernumerary teeth must be multidisciplinary. The management of the supernumerary teeth should form the part of a comprehensive treatment plan in cooperation with pediatric dentistry, oral surgeon, and orthodontists.

Leong's tubercle has to be evaluated in occlusal context and in cases of premature contact it has to be submitted into a selective grinding to avoid possible malocclusions and pulpal complications.
